# The Asthma Family Tree: Evaluating Associations Between Childhood, Parental, and Grandparental Asthma in Seven Chinese Cities

**DOI:** 10.3389/fped.2021.720273

**Published:** 2021-10-27

**Authors:** Hongyao Yu, Fan Su, Le-Bing Wang, Kari Hemminki, Shyamali C. Dharmage, Gayan Bowatte, Dinh Bui, Zhengmin Qian, Michael G. Vaughn, Hannah E. Aaron, Shimin Xiong, Xubo Shen, Yuanzhong Zhou, Peien Zhou, Xiao-Wen Zeng, Gongbo Chen, Bo-Yi Yang, Li-Wen Hu, Guang-Hui Dong

**Affiliations:** ^1^Department of Occupational and Environmental Health, Guangdong Provincial Engineering Technology Research Center of Environmental and Health Risk Assessment, School of Public Health, Sun Yat-sen University, Guangzhou, China; ^2^Faculty of Medicine and Biomedical Center in Pilsen, Charles University, Pilsen, Czechia; ^3^Division of Cancer Epidemiology, German Cancer Research Centre (DKFZ), Heidelberg, Germany; ^4^Allergy and Lung Health Unit, Melbourne School of Population and Global Health, University of Melbourne, Melbourne, VIC, Australia; ^5^Department of Epidemiology and Biostatistics, College for Public Health and Social Justice, Saint Louis University, Saint Louis, MO, United States; ^6^School of Social Work, College for Public Health and Social Justice, Saint Louis University, Saint Louis, MO, United States; ^7^Department of Epidemiology, School of Public Health, Zunyi Medical University, Zunyi, China

**Keywords:** childhood asthma, cross-sectional study, family history, hereditary patterns, mediation effect

## Abstract

**Objective:** To evaluate the associations between childhood, parental, and grandparental asthma.

**Methods:** We studied 59,484 children randomly selected from 94 kindergartens, elementary, and middle schools in seven Chinese cities from 2012 to 2013, using a cross-sectional survey-based study design. Information on their and their family members' (parents, paternal grandparents, and maternal grandparents) asthma status were reported by children's parents or guardians. Mixed effects logistic regressions were used to assess hereditary patterns of asthma and mediation analysis was performed to estimate the potential mediation effect of parents on the association between grandparental asthma and childhood asthma.

**Results:** The magnitude of ORs for childhood asthma increased as the number of family members affected by asthma increased. Among children who had one family member with asthma, childhood asthma was associated with asthma in maternal grandmothers (OR: 2.08, 95% CI: 1.67–2.59), maternal grandfathers (OR: 2.08, 95% CI: 1.71–2.53), paternal grandmothers (OR: 2.40, 95% CI: 1.93–2.99), and paternal grandfathers (OR: 2.59, 95% CI: 2.14–3.13). Among children who had two family members with asthma, the highest asthma risk was found when both parents had asthma (OR: 15.92, 95% CI: 4.66–54.45). Parents had a small proportion of mediation effect (9–12%) on the association between grandparental asthma and childhood asthma.

**Conclusions:** Grandparents with asthma were associated with childhood asthma and parents with asthma partially mediated the association.

## Introduction

One of the most common chronic diseases occurring among children is asthma. Asthma is characterized by shortness of breath, rapid breathing, chest tightness, and wheezing (1, 2). The prevalence of childhood has increased globally (3). In China, the prevalence of asthma in children increased from 0.91% in 1990 to 2.12% in 2010 (4). Asthma is one of the foremost reasons why children miss school days, visits the emergency department, and it can even affect a child's long-term health by impairing their lung function and increasing their risk of developing Chronic Obstructive Pulmonary Disease (COPD) (5). The etiology of childhood asthma is not fully understood. Thus, identifying its risk factors could increase early detection of the disease and prevent adverse outcomes later in life.

Asthma is genetically heritable with estimates derived from twin studies ranging widely from 35 to 70% (6). Although genome-wide research has identified a number of asthma susceptibility loci, e.g., 17q12-21(*ORMDL3, GSDMB*), 2q12 (*IL1RL1*/*IL18R1*), and 5q22 (*TSLP*) (7), they only account for a small fraction of the heritability and there are still genetic risk factors waiting to be unveiled (8, 9). As such, family history could provide useful information for gene susceptibility if shared environmental factors were controlled (10). Parents with asthma have been widely reported to contribute to increasing their offspring's asthma risk (11, 12). However, the relative roles of maternal and paternal asthma on their offspring's risk of asthma is controversial. Some studies found that maternal asthma had a stronger impact than paternal, whereas others had the opposite finding (13, 14). Importantly, grandparents are relatives who share 25% of a person's genes (15). Despite the usefulness of data on grandparental asthma, studies are relatively rare that have incorporated grandparental asthma into the risk equation for the etiology of childhood risk for asthma.

Given this research context, the aim of the present study was to investigate the multi-generational hereditary patterns of asthma. We estimated the familial risks of asthma for children who had parents, maternal grandparents, and paternal grandparents affected by asthma after controlling for potential environmental risk factors (e.g., passive smoke exposure, ambient air pollution, and pet kept in home) (16–18). In addition, we also estimated the role of parents' asthma on the association between childhood asthma and grandparental asthma.

## Methods

### Study Cities and Participants

Selection of the study cities and participants has been described previously in detail (19). In short, the cross-sectional study was conducted in 27 districts of seven northeast cities in China from 2012 to 2013, including six districts in Shenyang, three districts in Anshan, three districts in Benxi, five districts in Dalian, three districts in Dandong, four districts in Fushun, and three districts in Liaoyang. In each district, one or two kindergarten schools, primary schools, and middle schools were randomly selected (Supplementary Method 1). Totally, 94 kindergartens/schools were selected. In each grade of the selected school, one class was randomly chosen and all the students in the class were enrolled in the study. In total, we obtained 68,647 participants aged 0–20 years.

This study was carried out in strict accordance with the World Medical Association Declaration of Helsinki Ethical Principles for Medical Research Involving Human Subjects. The Human Studies Committee of Sun Yat-sen University approved the protocol. Before the study initiation, a signed informed consent was collected from all parents or guardians.

### Questionnaire

The information on doctor-diagnosed asthma among participants was collected by the Chinese version of the Epidemiologic Standardization Project Questionnaire of the American Thoracic Society (ATS-DLD-78-A), which was completed by parents or guardians. Doctor-diagnosed asthma was defined by the question “Has a doctor ever diagnosed this child with asthma?” In addition, asthma diagnoses for family members were also collected by the questions “Has this child's father ever had asthma?,” “Has this child's mother ever had asthma?,” “Has this child's paternal grandfather ever had asthma?,” “Has this child's paternal grandmother ever had asthma?,” “Has this child's maternal grandfather ever had asthma?,” and “Has this child's maternal grandmother ever had asthma?.” Based upon the total number of family members affected by asthma, participants were classified into four groups: no family member affected, 1 family member affected, 2 family members affected, and ≥ 3 family members affected.

### Covariates and Mediator

Information on covariates related to asthma was obtained from the questionnaire, including age (<5 years, 5–10 years, 10–15 years, and ≥15 years), gender (boy/girl), exercise time (hours per week), family income per year (<10,000 RMB, 10,000–29,999 RMB, 30,000–99,999 RMB, or ≥100,000 RMB), parental education (the highest educational level attained by either parent ≥ high school or lower), low birth weight (birth weight <2,500 g), premature birth (gestational age <37 weeks), breastfeeding (child being mainly breastfed for at least 3 months), obesity (yes/no), passive smoke exposure (child living with someone of the household who smokes daily at residence), home coal use (yes/no), pet kept (yes/no), and exposure to particulate matter with aerodynamic diameter <2.5 μm (PM_2.5_) (<48.97, 48.97–56.23, 56.23–60.57, or ≥60.57 μg/m^3^). The assessment of personal PM_2.5_ exposure has previously been described in detail (19, 20). The average PM_2.5_ concentration during 2009–2012, estimated based on each participant's residence using a machine learning method, was regarded as a surrogate of individual exposure (Supplementary Method 2). Participants were categorized into four groups based on quartiles of the PM_2.5_ concentration to which they were exposed. A directed acyclic graph (DAG) was drawn by the online tool DAGitty (http://dagitty.net/dags.html) (Supplementary Figure 1). Variables, containing passive smoke exposure, home coal use, pet kept. and PM_2.5_ exposure, were identified as potential confounders that needed adjusting in the model. In addition, having a parent with asthma was identified as a potential mediator.

### Statistical Analysis

The Chi-square test was used to compare categorical variables and *t*-test was used to compare continuous variables between asthma group and non-asthma group. Since the outcome (asthma in this study) was a dichotomous variable and participants from the same district tended to be similar, a mixed effects logistic regression was employed to estimate associations of different family history types with childhood asthma (Supplementary Method 3). Districts were considered as random effect. The odds ratios (ORs) and 95% confidence intervals (CIs) for participants who had 1, 2, or ≥ 3 family members affected were calculated after controlling for potential confounders, including passive smoke exposure, home coal use, pet kept and PM_2.5_ exposure. The reference group was participants who had no family members affected by asthma. Mediation effects of parents between grandparental asthma and childhood asthma were estimated after adjustment for potential confounders using R package mediation. The proportion mediated was the indirect effect divided by the total effect and 95% CIs were estimated using quasi-Bayesian approximation (100 simulations). In addition, a number of sensitivity analyses were conducted by adding covariates (age, sex, exercise time, family income per year, parental education, low birth weight, premature birth, breastfeeding, obesity) that are potentially related to asthma to the model. *P* < 0.05 was considered statistically significant. Data analyses were conducted using R version 3.6.3. The computation was run using the high-performance computer system in the School of Public Health at Sun Yat-sen University.

## Results

Among 68,647 participants, 63,910 (93.1%) returned the study questionnaires and 59,484 (86.7%) questionnaires were valid. The average age of the final participants was 10.23 years, and 49.39% were girls (Table 1). The prevalence of childhood asthma was 7.77%. Of those 4,619 participants diagnosed with asthma, 585 cases (12.67%) had one family member affected by asthma, 73 cases (1.58%) had two affected family members and 13 cases (0.28%) had at least three affected family members.

**Table 1:** Characteristics of participants.

**Variables**	**Total** **(*n* = 59,484)**	**Asthma**		***P*-value**
		**Yes** **(*n* = 4,619)**	**No** **(*n* = 54,865)**	
	***N*%**	***N*%**	***N*%**	
Age {years [mean (SD)]}^*^	10.23 (3.92)	9.50 (3.82)	10.29 (3.92)	<0.001
<5 years	5,647 (9.49)	480 (10.39)	5,167 (9.42)	<0.001
5–10 years	18,171 (30.55)	1,778 (38.49)	16,393 (29.88)	
10–15 years	28,000 (47.07)	1,954 (42.30)	26,046 (47.47)	
≥15 years	7,666 (12.89)	407 (8.81)	7,259 (13.23)	
Exercise time per week {hours [mean (SD)]}^*^	6.56 (7.96)	6.15 (7.64)	6.59 (8.00)	<0.001
Gender ^*^				<0.001
Boys	30,105 (50.61)	2,845 (61.59)	27,260 (49.69)	
Girls	29,379 (49.39)	1,774 (38.41)	27,605 (50.31)	
Family income per year ^*^				<0.001
<10,000RMB^a^	12,385 (20.82)	1,001 (21.67)	11,384 (20.75)	
10,000–30,000RMB^b^	22,071 (37.10)	1,613 (34.92)	20,458 (37.29)	
30,000–100,000RMB^c^	20,915 (35.16)	1,604 (34.73)	19,311 (35.20)	
≥100,000RMB^d^	4,113 (6.91)	401 (8.68)	3,712 (6.77)	
Parental education < high school ^*^	15,877 (26.69)	1,149 (24.88)	14,728 (26.84)	0.004
Low birth weight ^*^	2,175 (3.67)	216 (4.68)	1,959 (3.57)	0.001
Premature delivery ^*^	3,199 (5.38)	440 (9.53)	2,759 (5.03)	<0.001
Breastfeeding ^*^	39,576 (66.53)	2,772 (60.01)	36,804 (67.08)	<0.001
Obesity ^*^	4,346 (7.31)	398 (8.62)	3,948 (7.20)	0.004
Passive smoking exposure^*^	27,698 (46.56)	2,506 (54.25)	25,192 (45.92)	<0.001
Home coal use	3,439 (5.78)	282 (6.11)	3,157 (5.75)	0.326
Pet kept in home^*^	6,810 (11.45)	648 (14.03)	6,162 (11.23)	<0.001
PM2.5 ^*^				<0.001
<48.97 μg/m^3^	14,853 (24.97)	744 (16.11)	13,257 (25.72)	
48.97–56.23 μg/m^3^	13,977 (23.50)	979 (21.20)	14,501 (23.69)	
56.23–60.57 μg/m^3^	15,721 (26.43)	1,220 (26.41)	12,998 (26.43)	
≥60.57 μg/m^3^	14,933 (25.10)	1,676 (36.28)	14,109 (24.16)	
Asthma family history ^*^				<0.001
No family member affected	55,641 (93.54)	3,948 (85.47)	51,693 (94.22)	
1 family member affected	3,529 (5.93)	585 (12.67)	2,944 (5.37)	
2 family members affected	279 (0.47)	73 (1.58)	206 (0.38)	
≥ 3 family members affected	35 (0.06)	13 (0.28)	22 (0.04)	

* *p < 0·05 for difference between children with asthma and non-asthma children using t-test or chi-square test.*

*RMB, Chinese Yuan; PM_2.5_, particulate matter with aerodynamic diameters <2.5 μm.*

a *< US $1600;*

b *US $1600–US $4800;*

c *US $4800–US $16000;*

d *≥US $16000*.

**Figure 1:**
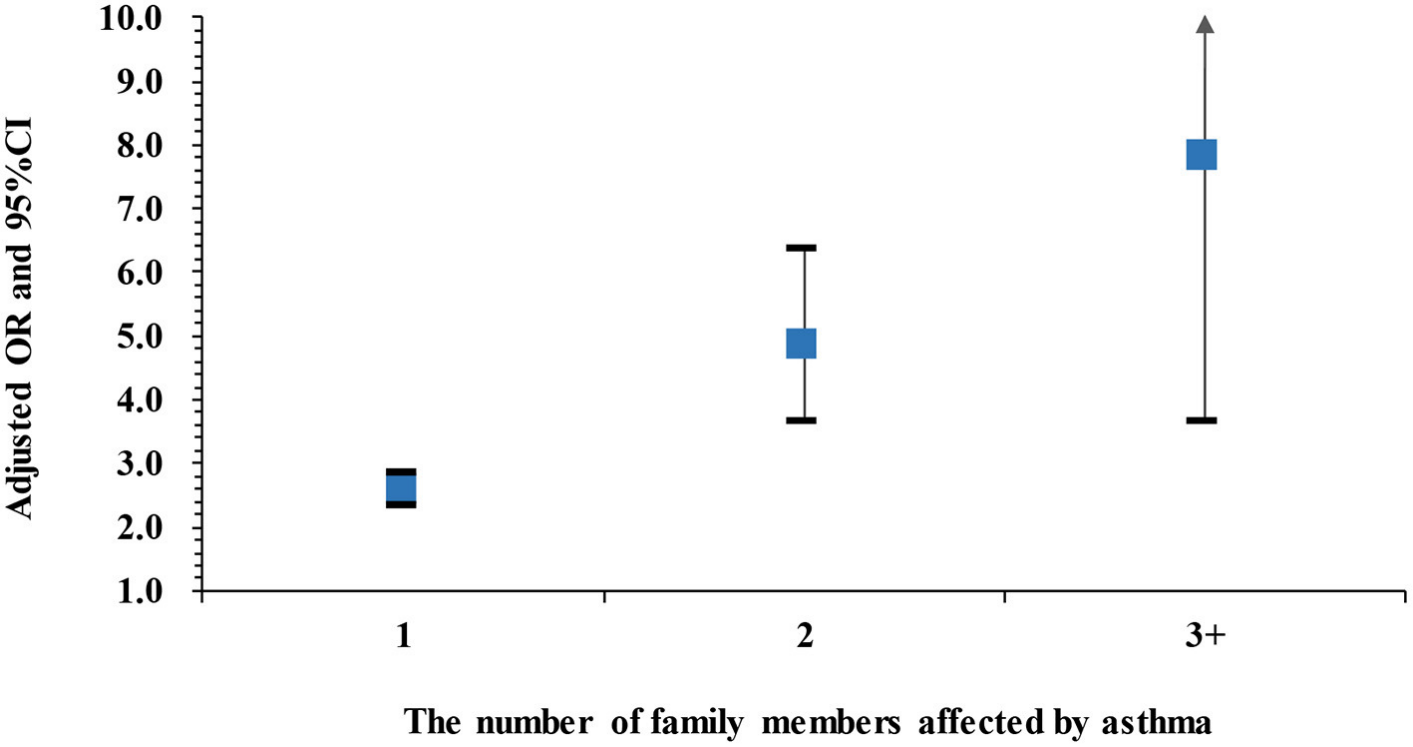
Adjusted ORs and 95%CIs for childhood asthma by the number of family members affected by asthma compared to those without any family member affected. Adjusted for passive smoke exposure, home coal use, pet kept and PM_2.5_ exposure. Asthma cases: 3,529 cases with 1 family member affected, 279 cases with 2 family members affected, and 35 cases with ≥3 family members affected.

Figure 1 shows the adjusted ORs for childhood asthma by the number of family members affected by asthma. The ORs were 2.58 (95% CI: 2.34–2.84), 4.63 (95% CI: 3.53–6.08), and 7.38 (95% CI: 3.70–14.73) when one, two and at least three family members were affected, respectively, and the trend was statistically significant (*P* < 0.001). The crude ORs that are shown in Supplementary Table 1 were slightly greater than the adjusted ORs.

**Figure 2:**
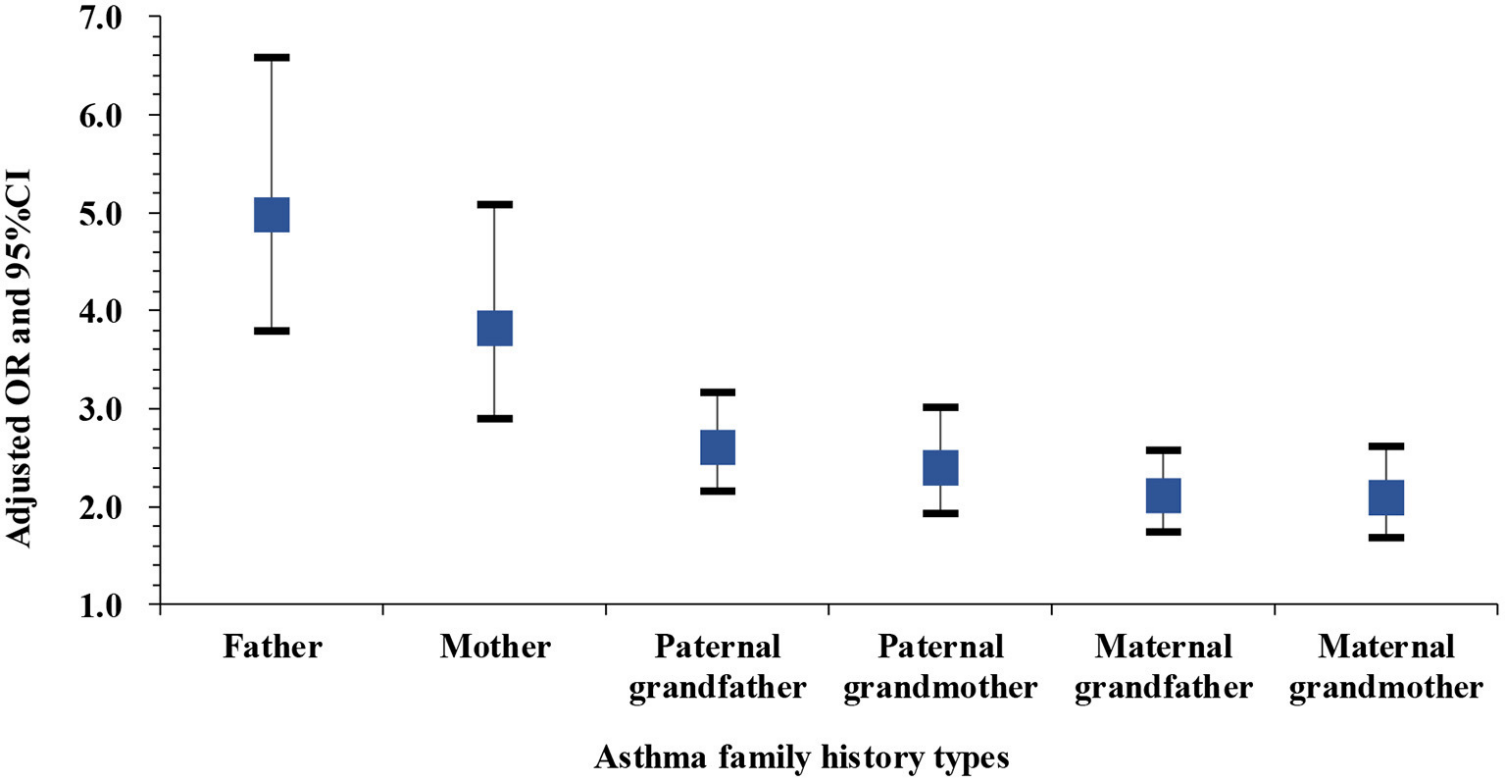
Adjusted ORs and 95%CIs for asthma among children with one family member affected by asthma compared to those without any family member affected. Adjusted for passive smoke exposure, home coal use, pet kept and PM_2.5_ exposure. Asthma cases: 75 cases with affected fathers, 67 cases with affected mothers, 132 cases with affected paternal grandfathers, 97 cases with affected maternal grandfathers, 119 cases with affected paternal grandmothers, and 95 cases with maternal grandmothers.

Among children with one family member affected by asthma, those with affected fathers had the highest adjusted OR (4.89, 95% CI: 3.72–6.42, Cases: 75), followed by those with affected mothers (OR: 3.94, 95% CI: 2.99–5.20, Cases: 67), paternal grandfathers (OR: 2.59, 95% CI: 2.14–3.13, Cases: 132), maternal grandfathers (OR: 2.40, 95% CI: 1.93–2.99, Cases: 97), paternal grandmothers (OR: 2.08, 95% CI: 1.71–2.53, Cases: 119), and maternal grandmothers (OR: 2.08, 95% CI: 1.67–2.59, Cases: 95) (Figure 2). The corresponding crude ORs are shown in Supplementary Table 2.

**Figure 3:**
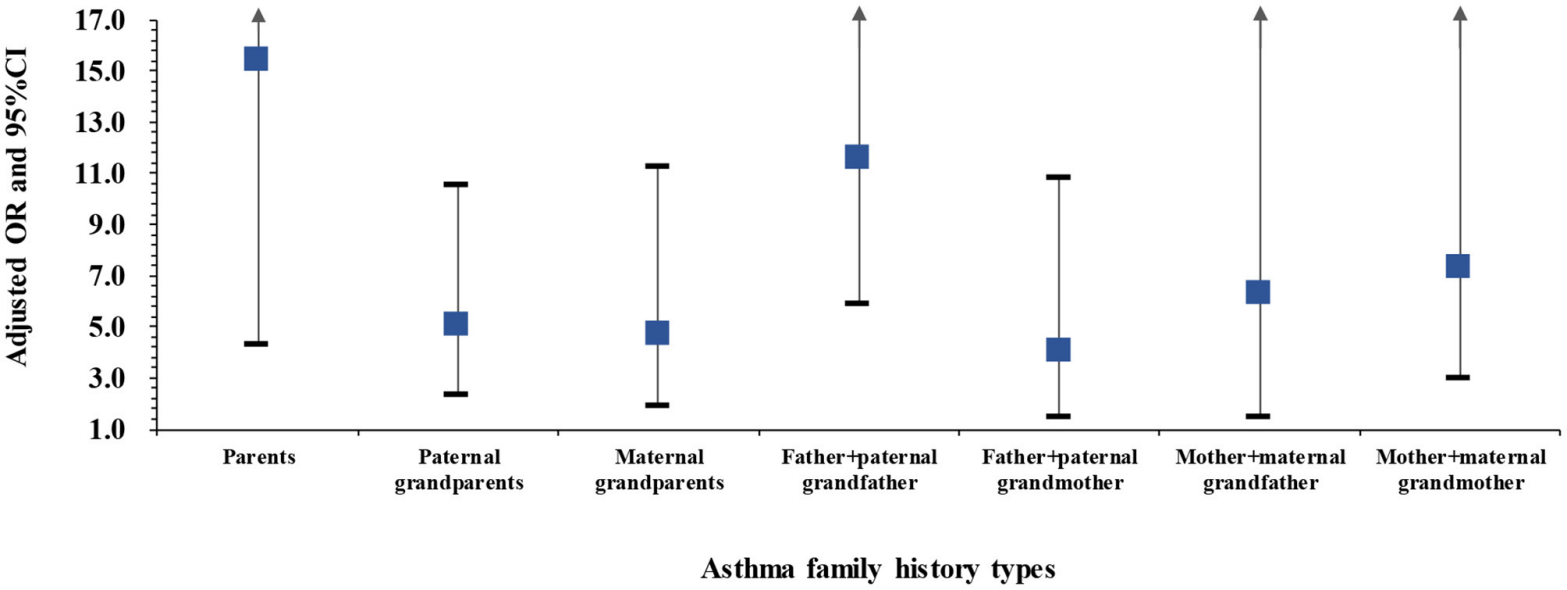
Adjusted ORs and 95%CIs for asthma among children with two family members affected by asthma compared to those without any family member affected. Adjusted for passive smoke exposure, home coal use, pet kept and PM_2.5_ exposure. Asthma cases: 5 cases with affected parents, 10 cases with paternal grandparents affected, 7 cases with maternal grandparents affected, 16 cases with father and paternal grandfather affected, 5 cases with father and paternal grandmother affected, 3 cases with mother and maternal grandfather affected, and 8 cases with mother and maternal grandmother affected.

Among children with two family members affected by asthma, the odds of asthma when both parents were affected (OR: 15.92, 95% CI: 4.66–54.45, Cases: 5) or when the father and paternal grandfather were affected (OR: 11.11, 95% CI: 5.77–21.38, Cases: 16) were higher than for other family history types, including paternal grandparents affected (OR: 5.04, 95% CI: 2.45–10.37, Cases: 10), maternal grandparents affected (OR: 4.39, 95% CI: 1.88–10.27, Cases: 7), father and paternal grandmother affected (OR: 3.64, 95% CI: 1.35–9.83, Cases: 5), mother and maternal grandfather affected (OR: 5.54, 95% CI: 1.46–21.07, Cases: 3), or mother and maternal grandmother affected (OR: 6.72, 95% CI: 2.86–15.81, Cases: 8) (Figure 3). The crude ORs shown in Supplementary Table 3 were slightly higher than the adjusted ORs.

After adjusted for passive smoke exposure, home coal use, pet kept, PM_2.5_ exposure, age, sex, exercise time, family income per year, parental education, low birth weight, premature birth, breastfeeding and obesity, the results of sensitivity analyses in Supplementary Tables 4–6 were generally consistent with the main findings.

Table 2 shows the proportion of the effect of grandparental asthma on their grandchildren's asthma that was mediated by parental asthma. Paternal asthma significantly mediated the association of childhood asthma with paternal grandfather asthma (12%, *P* < 0.001) and paternal grandmother asthma (11%, *P* < 0.001), and maternal asthma significantly mediated the association of childhood asthma with maternal grandfather asthma (6%, *P* < 0.001) and maternal grandmother asthma (9%, *P* < 0.001).

**Table 2:** Mediation effect of parental asthma between grandparental asthma and childhood asthma.

**Independent variable**	**Mediator**	**Proportion mediated**	**95%CI**	***P*-value**
Paternal grandfather	Father	12%	8–16%	<0.001
Paternal grandmother	Father	11%	7–18%	<0.001
Maternal grandfather	Mother	6%	3–9%	<0.001
Maternal grandmother	Mother	9%	5–14%	<0.001

*CI, confidence interval.*

*Adjusted for passive smoke exposure, home coal use, pet kept, and PM_2.5_ exposure*.

## Discussion

Relatively little is known regarding the multi-generational hereditary patterns of asthma. Taking advantage of a unique data source, we found that a family history of asthma was associated with a marked increase in the odds of childhood asthma and the magnitude of ORs increased as the number of affected family members enlarged. More specifically, independent of having a parent with asthma, a paternal grandparent or a maternal grandparent with asthma could increase the risk of children developing asthma. Children with a father and parental grandfather affected by asthma had less asthma risk than those with parents affected, but they had a higher risk than those with two other family members affected by asthma. In addition, we also identified that parental asthma partially mediated the associations between childhood asthma and grandparental asthma.

By combining family history of parents and grandparents affected by asthma, we observed that the magnitude of ORs for childhood asthma increased exponentially as the number of affected family members increased and the trend was statistically significant, suggesting that a combined family history could be used as a tool to predict the potential risk of a child developing asthma. A family history of a parent affected by asthma has been widely reported to increase asthma risk. For instance, a review of 33 studies found that the ORs of asthma for children with fathers affected by asthma ranged from 1.5 to 7.2, and for those with mothers affected ORs ranged from 1.5 to 9.7 (21). In our study, the ORs for a family history of father affected and mother affected were 4.89 and 3.94, respectively, which fell within the range observed in other studies.

It remains equivocal and somewhat controversial whether paternal or maternal asthma has a greater impact on the development of childhood asthma. We observed that children who had a father affected by asthma were at a greater risk than those with a mother affected. We also observed that although the ORs of asthma for those having a father and paternal grandfather with asthma were less than those having two parents with asthma, they were much higher than those who had two other family members with asthma, suggesting that inheritance of asthma tends to be paternally linked. The specific underlying biological mechanisms involved in these pathways is poorly understood. However, studies have identified that a family history of paternal asthma was associated with airway hyperresponsiveness (AHR) in children with asthma, whereas a family history of maternal asthma was not (22, 23). Since AHR is a major characteristic of asthma, it is speculated that genetic risk factors for AHR passed down from the father's side might contribute to childhood asthma (23). Nevertheless, as the number of the participants who had both father and grandfather with asthma was small in the present study, this finding needs to be replicated in larger samples.

A family history of second-degree relatives has been shown to be a robust risk factor for many diseases (24, 25), but studies investigating the impact of second-degree relatives on the development of childhood asthma are limited. A study on a Utah population found that a family history of second-degree relatives who died of asthma could increase the risk of asthma mortality by 34% (95% CI: 9–62%) (26). A previous study reported that, among 823 children diagnosed with asthma, 69.8% had a family history of asthma, of which 14.2% had an affected grandmother, 6.5% had an affected grandfather, and 3.3% had both affected grandparents (27). Valerio et al., who investigated intergenerational asthma, found that a family history of grandparental asthma was associated with childhood asthma and children with a parent and grandparent affected by asthma were at over four times greater risk of developing asthma compared to those with no parent and grandparent affected (28). Notably, these studies did not consider lineage (paternal grandparents or maternal grandparents), which have differential influences on certain diseases (29, 30). In our study, based on a sufficiently large sample, we were able to distinguish between grandparents of paternal and maternal lineage. Our results showed that a family history of either a paternal grandparent or a maternal grandparent having asthma was associated with an increased risk of childhood asthma independent of parental asthma, although the impact on the asthma risk was less than the impact of a family history of a parent having asthma. This finding suggests that shared asthma susceptibility genes play a small role in transgenerational inheritance. Furthermore, the results of our mediation analysis, which showed a small proportion of mediation effect of parental asthma, supports this observation. It is important to reiterate that asthma is a complex disease, and gene-gene and gene-environment interactions which influence asthma susceptibility may lead to the transgenerational inheritance of asthma from grandparents to grandchildren–in effect, skipping over the parents.

In our study, we recruited a large number of participants, which allowed us to differentiate paternal and maternal grandparents and to investigate the associations between family history of a grandparent with asthma and asthma risk in grandchildren. Environmental factors are obvious confounders for family history used to predict the hereditary patterns of a disease (10). In the present investigation, we controlled for a series of environmental factors associated with asthma, including PM_2.5_, a major ambient air pollution in recent years in China. Our study, however, has several limitations. Information on both childhood asthma and family history of asthma was obtained from self-reported questionnaires rather than hospital discharge registries, which may influence recall bias and misclassification of family history. Due to the one child-policy in China, the asthma diagnoses of siblings, which is an important genetic risk factor for a hereditary disease was left unevaluated and it may interpret the fact that the percentage of asthma cases who had family history in this study was relatively lower than that reported by Sheikh et al. (27). In addition, the small proportion of people who had at least two family members affected with asthma caused the large confidence intervals in Figures 1, 3. However, the lower 95%CIs for OR in each category were statistically higher than 1.

## Conclusions

In this study, we found that childhood asthma increased as the number of family members affected by asthma increased. A family history of a grandparent with asthma was associated with childhood asthma and the association was only partially mediated by a parent with asthma. In addition, children who had a father and a parental grandfather with asthma had a comparable magnitude of asthma risk to those who had both parents diagnosed with asthma. Although preliminary, our findings could be employed in a screening approach to identify children who may be a high risk of developing asthma in early childhood.

## Data Availability Statement

The raw data supporting the conclusions of this article will be made available by the authors, without undue reservation.

## Ethics Statement

The studies involving human participants were reviewed and approved by the Human Studies Committee of Sun Yat-sen University. Written informed consent to participate in this study was provided by the participants' legal guardian/next of kin.

## Author Contributions

G-HD and L-WH conceived of the presented idea. HY, FS, and L-BW developed the theory, performed the computations, and drafted the initial manuscript. KH, SD, GB, DB, ZQ, MV, HA, SX, XS, YZ, PZ, X-WZ, GC, and B-YY discussed the results and contributed to the final manuscript. All authors approved the final manuscript as submitted and agreed to be accountable for all aspects of the work.

## Funding

This study was supported by the National Key Research and Development Program of China (2018YFC1004300, 2018YFC1004302, and 2018YFE0106900), the National Natural Science Foundation of China (81872582 and 81872583), the Guangdong Provincial Natural Science Foundation Team Project (2018B030312005), the Science and Technology Program of Guangzhou (201807010032 and 201803010054), the Science and Technology Program of Zhongshan (2019B1110), and the Natural Science Foundation of Guangdong Province (2017A090905042, 2018B05052007, 2019A050510017, and 2020A1515011131). KH was sponsored by the European Union's Horizon 2020 Research and Innovation Programme, Grant no. 856620.

## Conflict of Interest

The authors declare that the research was conducted in the absence of any commercial or financial relationships that could be construed as a potential conflict of interest.

## Publisher's Note

All claims expressed in this article are solely those of the authors and do not necessarily represent those of their affiliated organizations, or those of the publisher, the editors and the reviewers. Any product that may be evaluated in this article, or claim that may be made by its manufacturer, is not guaranteed or endorsed by the publisher.
