# Supplementary material for: The Asthma Family Tree: Evaluating Associations Between Childhood, Parental, and Grandparental Asthma in Seven Chinese Cities
Source: Front Pediatr. 2021 Oct 27;9:720273. doi: 10.3389/fped.2021.720273 (PMC8579020; doi:10.3389/fped.2021.720273)
Supplement: Supplementary file 1 [file Data_Sheet_1.docx]

**Supplemental materials**

**The Asthma Family Tree: Evaluating Associations between Childhood, Parental, and Grandparental Asthma in Seven Chinese Cities.**

1. **METHODS**

**Method S1** Random sampling

**Method S2** PM_2.5_ assessment

**Method S3** Two-level logistic regression model

1. **RESULTS**

**Table S1.** Crude ORs and 95% CIs for childhood asthma by the number of family members affected

by asthma compared to those without any family member affected.

**Table S2.** Crude ORs and 95% CIs for asthma among children with one family member affected by asthma compared to those without any family member affected.

**Table S3.** Crude ORs and 95% CIs for asthma among children with two family members affected by asthma compared to those without any family member affected.

**Table S4.** Ajusted ORs and 95% CIs for childhood asthma by the number of family members affected

by asthma compared to those without any family member affected.

**Table S5.** Ajusted ORs and 95% CIs for asthma among children with one family member affected by asthma compared to those without any family member affected.

**Table S6.** Ajusted ORs and 95% CIs for asthma among children with two family members affected by asthma compared to those without any family member affected.

**Figure S1.** Directed acyclic graph for the association between grandparental asthma and childhood asthma.

1. **METHODS**

**Method S1 Random sampling**

In this study, 27 districts in seven cities in Liaoning Province in China were selected, including three in Liaoyang, Anshan, Dandong, and Benxi; four in Fushun; five in Dalian; and six in Shenyang. Schools in each district were marked with numbers.

Within one district, we randomly chose one or two middle schools, elementary schools, and kindergartens, respectively. In China, a district has a specific administrative code. In the study, we chose the last two non-zero numbers of the administrative code and used a random number table to determine which one or two elementary schools were randomly selected. For example, the code of Shenhe district in Shenyang is “210103”. Using our method, “03” from the code was selected. Correspondingly, the row of the random number table numbered “03” was chosen and the first number of this row “1” was taken as the start point. The school marked with “1” was enrolled into the study. If a student number less than 500 in a school, an extra school marked with “6” based on the second number of the row would be selected.

**Random number table**

**Method S2 PM_2.5_ assessment**

Daily ground-levels of PM_2.5_ and aerosol optical depth (AOD) were downloaded from the China National Environmental Monitoring Center (CNEMC) and NASA respectively. The Random Forest Model was developed to predict daily pollutant concentrations and the spatial resolution was 0.1^°^ (≈10 km). In the model, daily ground-level of PM_2.5_ was dependent variable, and AOD, meteorological and land-use data were independent **variables**. In the final model, the R^2^ (83%) and root-mean-squared error of 10-fold cross-validation (18.5μg/m^3^) for daily estimates of PM_2.5_ showed good predictive ability. Then we used this model to calculate PM_2.5_ concentrations. Addresses of the study sites were transformed into geocodes, the daily concentrations of PM_2.5_ from 2009 to 2012 were assessed using the model, and four-year average concentrations (2009-2012) were calculated as long-term exposure surrogates.

**Method S3 Two-level logistic regression model**

Since participants coming from the same district may be more similar, a 2-level logistic regression model was employed to assess the association between childhood, parental and grandparental asthma. Participants were the first-level unit, and the districts they lived were the second-level unit. For participant level, the model used to assess the log odds of asthma was as follows:

Y_ij_=β_0j_+β_1j_X_ij_ (1)

where the subscript i stands for the participant (i=1, ..., nj), the subscript j indicates the district (j=1, ..., 27), Y_ij_ is the log odds of having asthma for participant i in district j, β_0j_ is the intercept in district j, X_ij_ is a participant-level independent variable for participant i in district j and β_1j_ is the coefficient of X_ij_. For district level, the model with one district-level independent variable was as follows:

β_0j=_r_00_+r_01_W_j_+u_0j_ (2)

β_1j=_r_10_ (3)

where r_00_ is the overall intercept representing grand mean of the log odds of having asthma across all the districts, Wj is district-level independent variable for district j, r_01_ is the coefficient of Wj, u_0j_ is the error term of district-level representing a unique effect associated with district j, and r_10_ is the overall regression coefficient of X_ij_ at participant level.

Finally, a combined model obtained by substituting the values of β_0j_ and β_1j_ was as follows:

Y_ij_=r_00_+r_10_X_ij_+r_01_W_j_+u_0j_ (4)

As the combined model shown, the log odds of having asthma is affected by the overall log odds of having asthma across all the districts (r_00_), the effect of the participant-level (r_10_X_ij)_, district-level (r_01_W_j_) independent variables, and the district-level error (u_0j_). In our analysis, we used a random intercept-only model, where the random coefficient was assumed to be fixed and random intercepts were allowed to vary. Passive smoke exposure, home coal use, pet kept and PM_2.5_ exposure were added to the model as confounding variables. Odds ratios and 95% CI for childhood asthma by different asthma family histories were calculated.

1. **RESULTS**

**Table S1. Crude ORs and 95% CIs for childhood asthma by the number of family members affected**

**by asthma compared to those without any family member affected. (n=59484)**

| **The number of family members affected by asthma** |  | **OR** |  | **95%CI** |
| --- | --- | --- | --- | --- |
|  |  |  |  |  |
| **1** |  | 2.61 |  | (2.37-2.86) |
| **2** |  | 4.68 |  | (3.59-6.09) |
| **3^+^** |  | 7.83 |  | (3.98-15.41) |

Abbreviations: OR, odds ratio; CI, confidence interval.

**Table S2.** **Crude ORs and 95% CIs for asthma among children with one family member affected by asthma compared to those without any family member affected. (n=59170)**

| **Family member affected by asthma** |  | **OR** |  | **95%CI** |
| --- | --- | --- | --- | --- |
|  |  |  |  |  |
| **Father** |  | 5.04 |  | (3.86-6.59) |
| **Mother** |  | 4.04 |  | (3.07-5.30) |
| **Paternal grandfather** |  | 2.62 |  | (2.17-3.17) |
| **Paternal grandmother** |  | 2.41 |  | (1.94-3.00) |
| **Maternal grandfather** |  | 2.08 |  | (1.71-2.53) |
| **Maternal grandmother** |  | 2.10 |  | (1.69-2.61) |

Abbreviations: OR, odds ratio; CI, confidence interval.

**Table S3. Crude ORs and 95% CIs for asthma among children with two family members affected by asthma compared to those without any family member affected. (n=55808)**

| **Family members affected by asthma** |  | **OR** |  | **95%CI** |
| --- | --- | --- | --- | --- |
|  |  |  |  |  |
| Parents |  | 13.15 |  | (4.42-39.09) |
| Paternal grandparents |  | 4.94 |  | (2.49-9.82) |
| Maternal grandparents |  | 4.78 |  | (2.15-10.64) |
| Father and paternal grandfather |  | 10.47 |  | (5.72-19.17) |
| Father and paternal grandmother |  | 3.46 |  | (1.39-8.65) |
| Mother and maternal grandfather |  | 6.55 |  | (2.13-20.13) |
| Mother and maternal grandmother |  | 6.74 |  | (3.11-14.60) |

Abbreviations: OR, odds ratio; CI, confidence interval.

**Table S4. Adjusted ORs and 95% CIs for childhood asthma by the number of family members affected by asthma compared to those without any family member affected. (n=59484)**

| **The number of family members affected by asthma** |  | **OR** |  | **95%CI** |
| --- | --- | --- | --- | --- |
|  |  |  |  |  |
| **1** |  | 2.58 |  | (2.35-2.85) |
| **2** |  | 4.75 |  | (3.60-6.26) |
| **3^+^** |  | 7.32 |  | (3.60-14.92) |

Abbreviations: OR, odds ratio; CI, confidence interval.

Adjusted for passive smoke exposure, home coal use, pet kept, PM_2.5_ exposure, age, sex, exercise time, family income per year, parental education, low birth weight, premature birth, breastfeeding and obesity.

**Table S5.** **Adjusted ORs and 95% CIs for asthma among children with one family member affected by asthma compared to those without any family member affected. (n=59170)**

| **Family member affected by asthma** |  | **OR** |  | **95%CI** |
| --- | --- | --- | --- | --- |
|  |  |  |  |  |
| **Father** |  | 4.84 |  | (3.66-6.39) |
| **Mother** |  | 3.78 |  | (2.85-5.00) |
| **Paternal grandfather** |  | 2.65 |  | (2.18-3.21) |
| **Paternal grandmother** |  | 2.38 |  | (1.90-2.98) |
| **Maternal grandfather** |  | 2.10 |  | (1.72-2.57) |
| **Maternal grandmother** |  | 2.08 |  | (1.67-2.60) |

Abbreviations: OR, odds ratio; CI, confidence interval.

Adjusted for passive smoke exposure, home coal use, pet kept, PM_2.5_ exposure, age, sex, exercise time, family income per year, parental education, low birth weight, premature birth, breastfeeding and obesity.

**Table S6. Adjusted ORs and 95% CIs for asthma among children with two family members affected by asthma compared to those without any family member affected. (n=55808)**

| **Family members affected by asthma** |  | **OR** |  | **95%CI** |
| --- | --- | --- | --- | --- |
|  |  |  |  |  |
| Parents |  | 13.38 |  | (3.73-47.95) |
| Paternal grandparents |  | 5.01 |  | (2.37-10.59) |
| Maternal grandparents |  | 4.99 |  | (2.04-12.19) |
| Father and paternal grandfather |  | 11.81 |  | (6.05-23.05) |
| Father and paternal grandmother |  | 4.12 |  | (1.53-11.11) |
| Mother and maternal grandfather |  | 5.58 |  | (1.36-22.91) |
| Mother and maternal grandmother |  | 6.86 |  | (2.84-16.57) |

Abbreviations: OR, odds ratio; CI, confidence interval.

Adjusted for passive smoke exposure, home coal use, pet kept, PM_2.5_ exposure, age, sex, exercise time, family income per year, parental education, low birth weight, premature birth, breastfeeding and obesity.

**
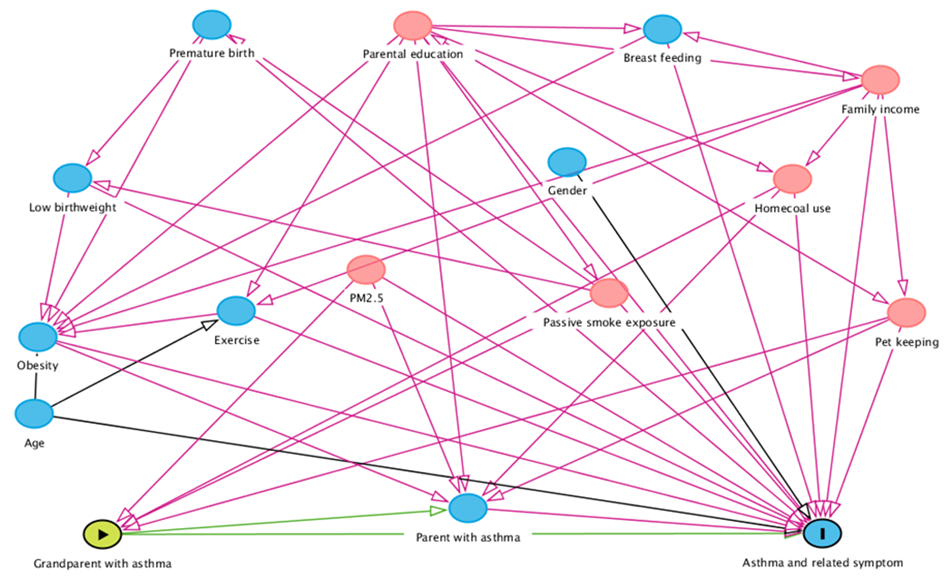
**

**Fig. S1. Directed acyclic graph for the association between grandparental asthma and childhood asthma.** Pink lines indicate biasing path, green lines indicate causal path and black lines indicate closed path.
